# 21-O-Angeloyltheasapogenol E3, a Novel Triterpenoid Saponin from the Seeds of Tea Plants, Inhibits Macrophage-Mediated Inflammatory Responses in a NF-*κ*B-Dependent Manner

**DOI:** 10.1155/2014/658351

**Published:** 2014-11-10

**Authors:** Woo Seok Yang, Jaeyoung Ko, Eunji Kim, Ji Hye Kim, Jae Gwang Park, Nak Yoon Sung, Han Gyung Kim, Sungjae Yang, Ho Sik Rho, Yong Deog Hong, Song Seok Shin, Jae Youl Cho

**Affiliations:** ^1^Department of Genetic Engineering, Sungkyunkwan University, Suwon 440-746, Republic of Korea; ^2^Medical Beauty Research Institute, AmorePacific R&D Center, Yongin 446-729, Republic of Korea

## Abstract

21-O-Angeloyltheasapogenol E3 (ATS-E3) is a triterpenoid saponin recently isolated from the seeds of the tea tree *Camellia sinensis* (L.) O. Kuntze. ATS-E3 has several beneficial properties including anti-inflammatory, antidiabetic, antiatherosclerotic, and anticancer effects. Unlike other phenolic compounds isolated from tea plants, there are no studies reporting the pharmacological action of ATS-E3. In this study, we therefore aimed to explore the cellular and molecular inhibitory activities of ATS-E3 in macrophage-mediated inflammatory responses. ATS-E3 remarkably diminished cellular responses of macrophages such as FITC-dextran-induced phagocytic uptake, sodium nitroprusside- (SNP-) induced radical generation, and LPS-induced nitric oxide (NO) production. Analysis of its molecular activity showed that this compound significantly suppressed the expression of inducible NO synthase (iNOS), nuclear translocation of nuclear factor- (NF-) *κ*B subunits (p50 and p65), phosphorylation of inhibitor of *κ*B kinase (IKK), and the enzyme activity of AKT1. Taken together, the novel triterpenoid saponin compound ATS-E3 contributes to the beneficial effects of tea plants by exerting anti-inflammatory and antioxidative activities in an AKT/IKK/NF-*κ*B-dependent manner.

## 1. Introduction

Macrophages are representative immune cells regulating the inflammatory barrier, an important defensive system against infecting pathogens such as bacteria, viruses, and fungi [1, 2]. To produce their inflammatory responses, these cells use special surface receptors (e.g., toll-like receptors (TLRs)) that are involved in recognizing biomaterials derived from pathogens and act as central molecules for managing macrophage functions during immunopathological processes. The activation of these receptors also transduces a variety of intracellular signaling cascades composed of phosphatidylinositide 3-kinase (PI3K), phosphoinositide-dependent kinase-1 (PDK1), AKT, inhibitor of *κ*B (I*κ*B) kinase (IKK), and I*κ*B*α*. These signaling pathways are linked to the nuclear translocation of transcription factors such as nuclear factor- (NF-) *κ*B to synthesize new inflammation-regulatory genes such as cytokines and chemokines. At the same time, macrophages produce a large number of toxic chemicals such as nitric oxide (NO) and reactive oxygen species (ROS) to directly attack and phagocytose infected pathogens in order to decrease the numbers of infected microorganisms. Although the macrophage-mediated inflammatory barrier is a useful biological event, functions that are overactivated by these cells can cause additional pathophysiological phenomena leading to immunological diseases. Prolonged inflammatory responses by macrophages are reported to induce loss of function in certain tissues or organs, leading to serious diseases including cancer, atherosclerosis, autoimmune diseases, and Alzheimer's disease. Therefore, suppression of acute or chronic inflammation is regarded as an acceptable therapeutic approach for the purpose of prevention or amelioration of diseases [3–7].

**Figure 1:**
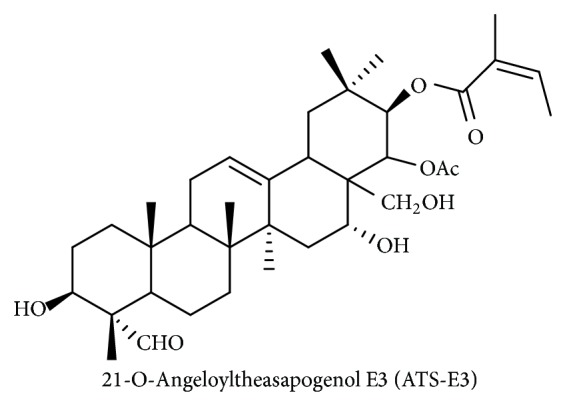
Chemical structure of ATS-E3.

To date, more than 1,800 papers have been published on tea plants (*Camellia sinensis* (L.) O. Kuntze), since tea is a very popular beverage with high levels of phenolic compounds. Unlike coffee, most studies on tea pharmacology have stressed the beneficial roles of these plants including neuroprotective, hepatoprotective, antioxidative, antidiabetic, anticancer, antiobesity, antibacterial, anticardiovascular, antilipogenic, and antiaging activities. Such desirable activities have led scientists to study active components producing these various pharmacological activities. Consequently, numerous chemicals such as catechins, theaflavins, tannins, and flavonoids have been identified from the leaves, seeds, or roots of tea trees. Previous reports suggest that polyphenolic compounds may be pharmacologically valuable components. In contrast, only a few papers have been published on triterpenoid saponins from tea plants. The pharmacological effects of olean-12-ene-type triterpenoid saponins from the roots of tea plants are unknown [8]. Oleiferasaponin A_1_, which has antiapoptotic activity, has been isolated from the tea seed pomace (*Camellia oleifera* Abel) [9]. Considering that saponin fractions from* Panax ginseng* and* Codonopsis lanceolata* have been proposed as nontoxic anti-inflammatory remedy and cosmetic sources, we also aimed to isolate triterpenoid saponins with anti-inflammatory properties. Therefore, in this study, we report a novel triterpenoid saponin, 21-O-angeloyltheasapogenol E3 (ATS-E3), (Figure 1) isolated from tea plant seeds and its anti-inflammatory activity in macrophage-mediated inflammatory responses.

## 2. Materials and Methods

### 2.1. Materials

Sodium nitroprusside (SNP), 3-(4,5-dimethylthiazol-2-yl)-2,5-diphenyltetrazolium bromide (MTT), dihydrorhodamine 123 (DHR123), fluorescein isothiocyanate- (FITC-) dextran, N*ω*-nitro-L-arginine methyl ester (L-NAME), and lipopolysaccharide (LPS;* E. coli* 0111:B4) were purchased from Sigma Chemical Co. (St. Louis, MO, USA). BAY11-7082 (BAY), LY294002, and wortmannin were obtained from Calbiochem (La Jolla, CA, USA). Fetal bovine serum and RPMI 1640 were obtained from Gibco (Grand Island, NY, USA). The murine macrophage cell line RAW264.7 and human embryonic kidney (HEK) 293 cells were purchased from the American Type Culture Collection (Rockville, MD, USA). All other chemicals were of analytical grade and were obtained from Sigma. A luciferase construct containing binding sites for NF-*κ*B was a gift from Professor Hae Young Chung (Pusan National University, Pusan, Korea). DNA constructs with FLAG-MyD88 and Myc-Syk were used as reported previously [10]. Phosphospecific and/or total antibodies against NF-*κ*B subunits (p50 and p65), I*κ*B*α*, IKK, AKT, PDK1, lamin A/C, and *β*-actin were obtained from Cell Signaling (Beverly, MA, USA).

### 2.2. Preparation of 21-O-Angeloyltheasapogenol E3

21-O-Angeloyltheasapogenol E3 (ATS-E3) was prepared from the ethanolic extract of the seeds of tea plants. The extract was partitioned between ethyl acetate and distilled water, and then the ethyl acetate layer was dried to yield the ethyl acetate fraction. ATS-E3 was purified from the ethyl acetate fraction using MPLC (under a hexane-ethyl acetate solvent gradient) and was isolated as a white solid. Its physicochemical and spectroscopic data (see supplementary Figures 1 and 2 in Supplementary Material available online at http://dx.doi.org/10.1155/2014/658351) were the same as those of published values [11–13]. The seeds of tea plants used in this study were obtained from Cheju Island in Korea.

### 2.3. Cell Culture

RAW264.7 and HEK293 cells were cultured in RPMI 1640 medium supplemented with 10% heat-inactivated fetal bovine serum (FBS; Gibco, Grand Island, NY, USA), glutamine, and antibiotics (penicillin and streptomycin) at 37°C under 5% CO_2_. For each experiment, the cells were detached with a cell scraper. When the cells were cultured for the experiments at a density of 2 × 10^6^ cells/mL, the proportion of dead cells was less than 1% as determined by Trypan blue dye exclusion.

### 2.4. Determination of Phagocytic Uptake

To measure the phagocytic activity of RAW264.7 cells, we modified a previously reported method [14]. RAW264.7 cells (5 × 10^4^) were pretreated with ATS-E3 (0 to 10 *μ*M) or BAY11-7082 (10 and 15 *μ*M) for 1 h and then resuspended in 100 *μ*L phosphate-buffered saline (PBS) containing 1% human AB serum and incubated with FITC-dextran (1 mg/mL) at 37°C for 20 min. The reactions were stopped by adding 2 mL of ice-cold PBS containing 1% human serum and 0.02% sodium azide. The cells were then washed three times with cold PBS-azide and analyzed on a FACScan flow cytometer (Becton-Dickinson, San Jose, CA, USA) as reported previously [15].

### 2.5. Determination of Reactive Oxygen Species Generation

The level of intracellular ROS was determined by recording the change in fluorescence resulting from the oxidation of the fluorescent probe DHR123. Briefly, 5 × 10^5^ RAW264.7 cells were exposed to ATS-E3 (0 to 10 *μ*M) for 30 min and then incubated with SNP (0.25 mM) at 37°C for 20 min to induce ROS production. The cells were further incubated with 20 *μ*M of the fluorescent probe DHR123 for 30 min at 37°C. The degree of fluorescence, which corresponded to the level of intracellular ROS, was determined using a FACScan flow cytometer (Becton-Dickinson) as reported previously [15].

### 2.6. Flow Cytometric Analysis

The level of FITC-dextran or ROS in RAW264.7 cells was determined by flow cytometric analysis [16, 17]. RAW264.7 cells (2 × 10^6^ cells/mL) treated with AP736 in the presence or absence of FITC-dextran (1 mg/mL) or DHR123 were washed with staining buffer containing 2% rabbit serum and 1% sodium azide in PBS and incubated with directly labeled antibodies for an additional 45 min on ice. After washing three times with staining buffer, stained cells were analyzed on a FACScan flow cytometer (Becton-Dickinson).

### 2.7. NO Production

RAW264.7 macrophage cells (1 × 10^6^ cells/mL) were cultured for 18 h, pretreated with ATS-E3 (0 to 10 *μ*M) for 30 min, and further incubated with LPS (1 *μ*g/mL) for 24 h. The inhibitory effect of ATS-E3 on LPS-induced NO production was determined by analyzing NO level using Griess reagent as previously described [18, 19]. The OD at 550 nm (OD_550_) was measured using a SpectraMax 250 microplate reader (Molecular Devices, Sunnyvale, CA, USA).

### 2.8. Cell Viability Test

RAW264.7 cells (1 × 10^6^ cells/mL) were cultured for 18 h, after which ATS-E3 (0 to 10 *μ*M) was added to the cells for the final 24 or 8 h of culture, respectively. The cytotoxic effect of ATS-E3 was then evaluated by a conventional MTT assay as reported previously [20, 21]. For the final 3 h of culture, 10 *μ*L MTT solution (10 mg/mL in PBS, pH 7.4) was added to each well. The incubation was stopped by the addition of 15% sodium dodecyl sulfate (SDS) into each well, which solubilized the formazan [22]. The absorbance at 570 nm (OD_570–630_) was measured using a SpectraMax 250 microplate reader (BioTek, Bad Friedrichshall, Germany).

### 2.9. Analysis of iNOS Expression by Real-Time Reverse Transcription-Polymerase Chain Reaction

RAW264.7 cells (1 × 10^6^ cells/mL) were cultured for 18 h, pretreated with ATS-E3 (0 to 10 *μ*M) for 30 min, and further cultured with LPS (1 *μ*g/mL) for 6 h. The inhibitory effect of ATS-E3 on the expression of iNOS was determined by real-time quantitative reverse transcription-polymerase chain reaction (qRT-PCR) [18, 23]. To determine the level of iNOS gene expression, total RNA was isolated from LPS-treated RAW264.7 cells using TRIzol Reagent (Gibco BRL) according to the manufacturer's instructions. The total RNA was stored at −70°C until use. Real-time qRT-PCR was performed as reported previously [24, 25]. The primers (Bioneer, Daejeon, Korea) used in these reactions are listed in Table 1.

### 2.10. Plasmid Transfection and Luciferase Reporter Gene Activity Assay

HEK293 cells (1 × 10^6^ cells/mL) were transfected with 1 *μ*g of plasmids driving the expression of *β*-galactosidase and either NF-*κ*B-Luc or AP-1-Luc in the presence or absence of an inducing molecule MyD88 or tyrosine kinase Myc-Syk. Transfections were performed using the PEI method in 12-well plates as previously outlined [26, 27]. Transfected cells were used at 48 h posttransfection for all experiments. Cells were treated with ATS-E3 for the final 8 h of each experiment. Luciferase assays were performed using the Luciferase Assay System (Promega, Madison, WI), as previously reported [28].

### 2.11. Preparation of Cell Lysates and Immunoblotting Analysis

RAW264.7 cells (5 × 10^6^ cells/mL) were washed three times in cold PBS supplemented with 1 mM sodium orthovanadate, resuspended in lysis buffer (20 mM Tris-HCl, pH 7.4, 2 mM EDTA, 2 mM ethyleneglycotetraacetic acid, 50 mM *β*-glycerophosphate, 1 mM sodium orthovanadate, 1 mM dithiothreitol, 1% Triton X-100, 10% glycerol, 10 *μ*g/mL aprotinin, 10 *μ*g/mL pepstatin, 1 mM benzamide, and 2 mM PMSF), lysed by sonication, and rotated for 30 min at 4°C. The lysates were clarified by centrifugation at 16,000 ×g for 10 min at 4°C and stored at −20°C until use. The soluble fractions of the cell lysates were immunoblotted, and total and phosphoprotein levels of p50, p65, I*κ*B*α*, IKK, AKT, PDK1, lamin A/C, and *β*-actin were visualized as previously reported [29].

### 2.12. PDK1 and AKT1 Kinase Assay

To evaluate the ability of ATS-E3 to inhibit the activity of purified PDK1 and AKT1, we used the Millipore Kinase Profiler service (Billerica, MA, USA) as reported previously [30]. Human PDK1 or AKT1 (1–5 mU) was incubated in reaction buffer in a final reaction volume of 25 *μ*L. The reaction was initiated by the addition of MgATP. After incubation for 40 min at room temperature, the reaction was stopped by the addition of 5 mL of a 3% phosphoric acid solution. The reaction product (10 *μ*L) was spotted onto a P30 Filtermat and washed three times for 5 min each with 75 mM phosphoric acid and once in methanol prior to drying and scintillation counting.

### 2.13. Statistical Analysis

Data are expressed as the mean ± standard deviation (SD), as calculated from one (*n* = 6) of two independent experiments. Other data are representative of three different experiments with similar results. For statistical comparisons, the results were analyzed using analysis of variance/Scheffe's post-hoc test and the Kruskal-Wallis/Mann-Whitney test. A *P* value < 0.05 was considered to be statistically significant. All statistical tests were conducted using SPSS (SPSS Inc., Chicago, IL, USA).

## 3. Results and Discussion

**Figure 2:**
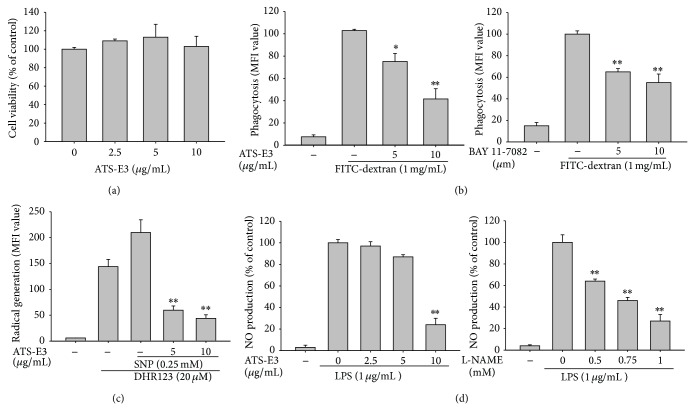
The effects of ATS-E3 on macrophage-mediated inflammatory responses. (a) RAW264.7 cells (1 × 10^6^ cells/mL) were treated with ATS-E3 for 6 or 24 h. Cell viability was evaluated using the MTT assay. (b) RAW264.7 cells preincubated with ATS-E3 (left panel) or BAY11-7082 (right panel) were treated with FITC-dextran (1 mg/mL) for 2 h. The level of dextran uptake was determined by flow cytometric analysis. (c) Scavenging effect of ATS-E3 on reactive oxygen species (ROS) generation in sodium nitroprusside- (SNP-) treated RAW264.7 cells was examined by flow cytometric analysis using DHR123 (20 *μ*M) and SNP (0.25 mM). (d) NO inhibitory activities of ATS-E3 (left panel) or L-NAME (right panel) were determined with RAW264.7 cells (1 × 10^6^ cells/mL) treated with LPS (1 *μ*g/mL) in the presence or absence of ATS-E3 or L-NAME for 24 h. The cell culture supernatants were collected, and the concentrations of NO in the supernatants were determined using the Griess assay. ^*^ *P* < 0.05 and ^**^ *P* < 0.01 compared to control group.

No previous studies have reported the biological activity of ATS-E3, a novel triterpenoid saponin isolated from* Camellia sinensis*; therefore, we aimed to evaluate its pharmacological activity under macrophage-mediated inflammatory conditions. To start this study, we first examined the cytotoxic activity of ATS-E3 in macrophage-like RAW264.7 cells. As Figure 2(a) shows, there was no significant toxic effect affecting cell viability under the treatment of this compound up to 10 *μ*M, indicating that the activity of ATS-E3 at 0 to 10 *μ*M concentration ranges could be due to its specific pharmacological activities.

During innate immune responses, the primary function of macrophages is to phagocytose infected or exogenous materials. Such phagocytic ability can be easily mimicked with macromolecules labeled with FITC. In fact, FITC-dextran showed a 10-fold increase in RAW264.7 macrophage-like cells (Figure 2(b)), as assessed by flow cytometric analysis in a previous report [31]. Interestingly, ATS-E3 blocked phagocytosis of FITC-dextran in a dose-dependent manner (Figure 2(b), left panel). Moreover, the IKK inhibitor BAY 11-7082 also significantly diminished the uptake of FITC-dextran, indicating that phagocytosis of FITC-dextran by macrophages is dependent on IKK/NF-*κ*B (Figure 2(b), right panel), as reported previously [32]. Additional functions of macrophages include the release of large amounts of toxic molecules such as ROS and RNS, which cause cellular damage in tissues or organs and lead to loss of function due to inflammation [33]. Therefore, we next examined whether ATS-E3 could neutralize toxic molecules by using the radical generator SNP. To produce antioxidative activity under cellular conditions, we also used RAW264.7 cells treated with SNP. As shown in Figure 2(c), this compound completely neutralized the generated radicals. Since triterpenoid saponins such as parkioside B from the root bark of* Butyrospermum parkii* [34], 3*β*-O-*α*-l-arabinopyranosyl-19*α*,23-dihydroxy-20*α*-urs-12-en-28-oic acid 28-O-*β*-d-glucopyranosyl ester from the aerial parts of* Ilex cornuta* [35], and 3*β*-hydroxy-23-oxo-30-noroleana-12,20(29)-diene-28-oic acid 3-*O*-*β*-d-glucuronopyranosyl-28-*O*-*β*-d-glucopyranoside from* Salicornia herbacea* [36] display strong antioxidative activity, the scavenging action of ATS-E3 may be due to their phytochemical properties. Therefore, these results imply that ATS-E3 can downregulate phagocytosis and prevent the generation of radicals mediated by the functional activation of macrophages.

The fact that the IKK/NF-*κ*B inhibitor BAY suppressed FITC-dextran-induced phagocytosis in RAW264.7 cells (Figure 2(b), right panel) led us to hypothesize that ATS-E3 can block NF-*κ*B-dependent inflammatory responses. To determine this, we first tested the inhibitory activity of this saponin on NO production, a representative NF-*κ*B-dependent response [37], in LPS-treated RAW264.7 cells. As shown in the left panel of Figure 2(d), NO levels strongly decreased (up to 80%) at a concentration of 10 *μ*g/mL ATS-E3. However, there was no suppression of cell viability, according to the MTT assay (Figure 2(a)), implying that the inhibitory activity of ATS-E3 on NO production was not due to simple nonspecific cytotoxicity and that ATS-E3 can block NF-*κ*B-mediated macrophage functions. Meanwhile, the standard compound L-NAME exhibited dose-dependent inhibitory activity up to 1 mM (Figure 2(d), right panel), indicating that the experimental conditions were well established, as reported previously [38].

**Figure 3:**
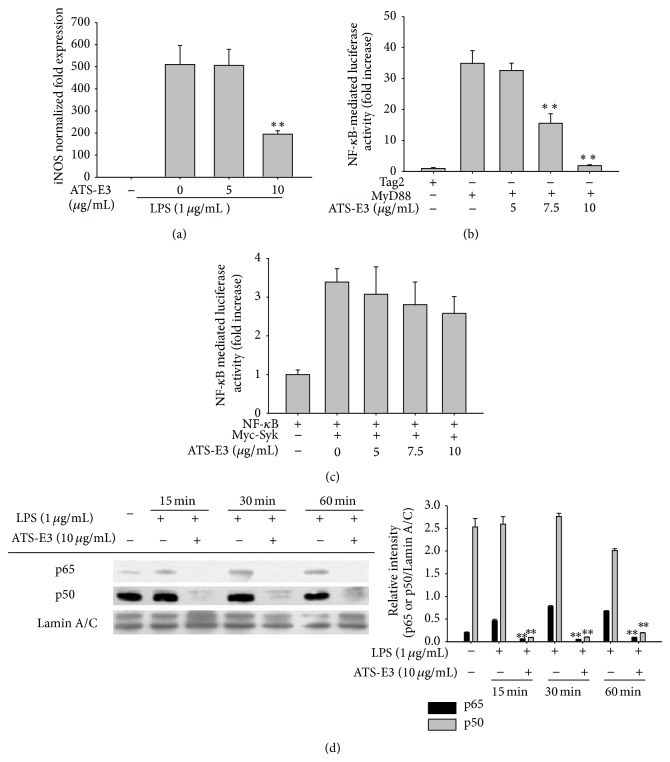
Effect of ATS-E3 on the transcriptional activation of RAW264.7 cells during TLR signaling. (a) The level of iNOS mRNA in RAW264.7 cells treated with ATS-E3 (0 to 10 *μ*M) in the presence or absence of LPS (1 *μ*g/mL) for 6 h was determined by real-time quantitative RT-PCR. ((b) and (c)) HEK293 cells cotransfected with NF-*κ*B-Luc and *β*-gal (as a transfection control) plasmid constructs were treated with ATS-E3 under the cotransfection conditions with FLAG-MyD88 or Myc-Syk (1 *μ*g/mL each) for 12 h. Luciferase activity was determined using luminometery, as described in Section 2. (d) RAW264.7 cells (5 × 10^6^ cells/mL) were incubated with LPS (1 *μ*g/mL) in the presence or absence of ATS-E3 for the indicated times. After preparing the nuclear fractions, the translocated levels of total transcription factors (p65, p50, and lamin A/C) were identified using immunoblotting. Relative intensity was calculated using total levels by the DNR Bio-Imaging System. ^**^ *P* < 0.01 compared to control group.

To obtain further evidence of the suppression of NF-*κ*B-dependent inflammatory events, transcriptional activation and nuclear translocation of NF-*κ*B were examined by measuring mRNA level of iNOS and performing the luciferase reporter gene assay for nuclear levels of NF-*κ*B subunits (p50 and p65). As depicted in Figure 3(a), the level of iNOS was remarkably diminished by 10 *μ*g/mL of ATS-E3. In addition, NF-*κ*B-mediated luciferase activity induced by MyD88 (Figure 3(b)) but not Syk (Figure 3(c)), a NF-*κ*B-activating upstream tyrosine kinase [39], was dose-dependently inhibited by this saponin. The nuclear translocation levels of NF-*κ*B subunits were also clearly reduced in response to ATS-E3 treatment (10 *μ*g/mL) at 15, 30, and 60 min (Figure 3(d), left and right panels). These results strongly support our hypothesis regarding NF-*κ*B as a target transcription factor of ATS-E3. Considering that several triterpenoid saponins have also been reported to suppress NF-*κ*B activation [40, 41], it is possible that a structural unit in triterpenoid saponins contributes to the inhibition of NF-*κ*B activation.

**Figure 4:**
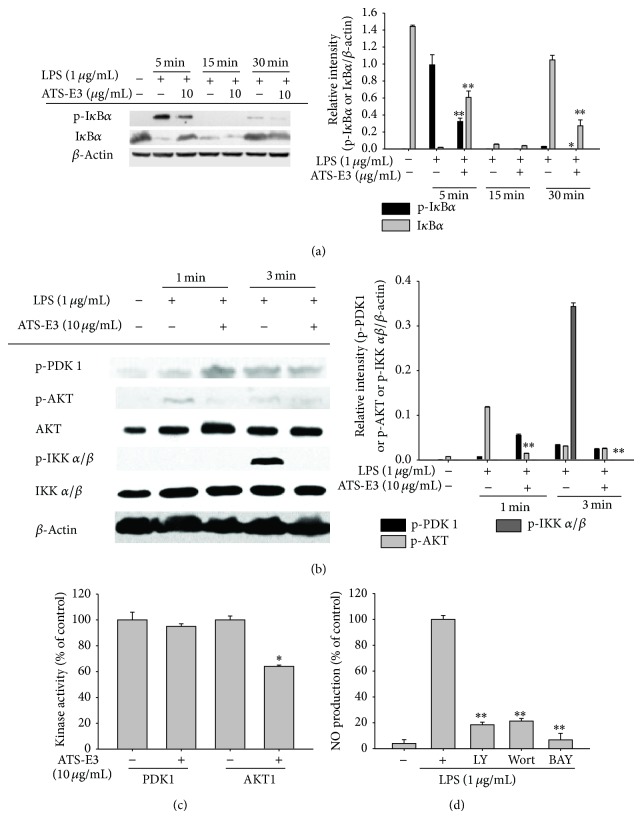
Effect of ATS-E3 on the activation of NF-*κ*B upstream signaling cascades. ((a) and (b)) Total and phosphoprotein levels of PDK1, AKT, IKK*α*/*β*, and *β*-actin in whole cell lysates of LPS-treated RAW264.7 cells were determined by immunoblotting analysis. (c) Kinase activities of PDK1 and AKT1 were determined by a direct kinase assay using purified enzymes. The value of the control, which received vehicle treatment, was set as 100% activity for each enzyme (PDK1 or AKT1). (d) NO inhibitory activities of standard compounds (BAY11-7082 (BAY), LY294002 (LY), and wortmannin (Wort)) were determined in RAW264.7 cells (1 × 10^6^ cells/mL) treated with LPS (1 *μ*g/mL) in the presence or absence of standard compounds for 24 h. Relative intensity was calculated using total levels by the DNR Bio-Imaging System. ^*^ *P* < 0.05 and ^**^ *P* < 0.01 compared to control group.

Finally, since the translocation of NF-*κ*B subunits was inhibited by ATS-E3, we next explored a potential target enzyme involved in regulating the translocation of NF-*κ*B. Interestingly, I*κ*B*α* phosphorylation, a critical step for translocation of NF-*κ*B subunits [42], was strongly reduced at 5 and 30 min (Figure 4(a), left and right panels), implying that the ATS-E3-targeting enzyme was activated at early time points. Therefore, we next evaluated continuous phosphorylation levels of upstream enzymes participating in I*κ*B*α* phosphorylation at 5 min by preparing whole lysates prepared from RAW264.7 cells stimulated by LPS exposure for 1 and 3 min. As shown in Figure 4(b) (left and right panels), early phosphorylation of IKK and AKT at 1 and 3 min was clearly reduced by this compound, whereas the phosphorylation of PDK1 was not suppressed, indicating that PDK1 is a potential target of ATS-E3. To study this possibility, we conducted a kinase assay with purified PDK1 and AKT1. Although we failed to observe strong inhibitory activity by ATS-E3, we found that ATS-E3 (10 *μ*g/mL) significantly suppressed AKT1 kinase activity up to 40% (Figure 4(c)). In contrast, PDK1 activity was not reduced by this compound (Figure 4(c)), suggesting that it can directly suppress the enzyme activity of AKT, known as IKK-phosphorylating enzyme [43]. Evidence suggests that AKT plays a positive role in NF-*κ*B-mediated inflammatory responses. In the present study, overexpression of AKT resulted in IKK phosphorylation, I*κ*B*α* degradation, and NF-*κ*B translocation. Also, AKT inhibition by LY294002 and wortmannin suppressed the production of inflammatory mediators (Figure 4(d)). Indeed, we also confirmed that these compounds as well as the IKK inhibitor BAY 11-7082 were capable of reducing the production of NO under LPS stimulation (Figure 4(d)). In addition, we recently found that Cys310 of AKT plays an important proinflammatory role [44]. Indeed, compounds that can bind to the thiol group of this cysteine residue clearly show anti-inflammatory properties by suppressing the NF-*κ*B pathway [44–46]. However, the finding that ATS-E3 blocked only 40% of the AKT activity (Figure 4(c)) indicates that there is another major target in ATS-E3 pharmacology. Therefore, future studies should include identification of ATS-E3 target proteins. Taken together, previous reports and our data strongly suggest positive regulation of inflammatory pathways, and AKT/IKK/NF-*κ*B regulatory loops are directly targeted by the anti-inflammatory activity of ATS-E3.

**Figure 5:**
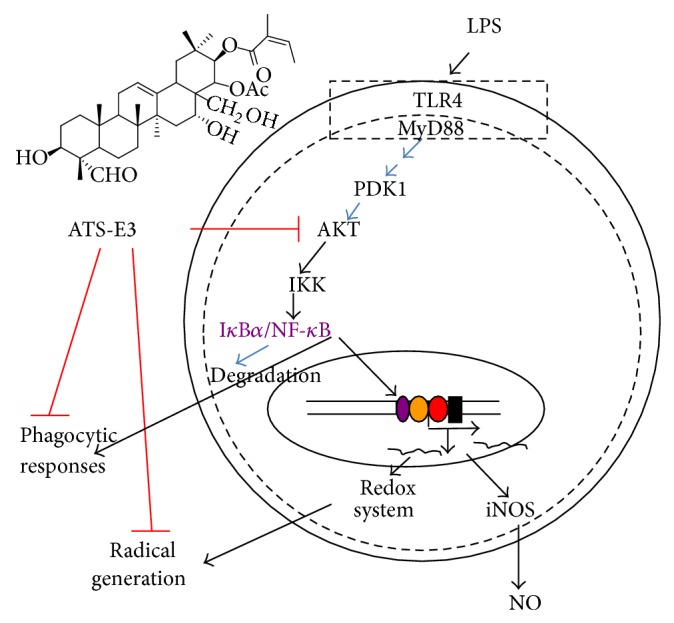
Putative inhibitory pathway of macrophage-mediated inflammatory responses by ATS-E3.

In summary, ATS-E3 strongly inhibited macrophage-mediated inflammatory responses such as phagocytic uptake, ROS generation, and NO production. The inhibitory action of ATS-E3 was mediated by suppression of inflammatory pathways composed of AKT, IKK, and NF-*κ*B, as summarized in Figure 5. Therefore, our findings strongly suggest that the novel triterpenoid saponin component ATS-E3 may contribute to the beneficial role of tea plants through its anti-inflammatory activity. In view of the present data, we also propose that ATS-E3 can be further developed as the first anti-inflammatory saponin component prepared from tea plants. Therefore, our future studies will focus on providing additional pharmacological evidence to demonstrate this possibility.

## Supplementary Material

Supplementary Figure 1: ^1^H NMR spectra were recorded on a Bruker Avance 300 (300 MHz) and Bruker DPX 400 (400 MHz). Chemical shifts are reported in parts per million (ppm) downfield relative to tetramethylsilane as an internal standard.
Supplementary Figure 2: ESI-MS was measured on an Agilent 1100 LC/MS spectrometer with a Phenomenex Luna C18 analytical column (5 mm, 4.6 × 100 mm).

## Figures and Tables

**Table 1:** Primer sequences used for real-time PCR analysis.

Name		Sequence (5′ to 3′)
iNOS	F	CCCTTCCGAAGTTTCTGGCAGCAG
	R	GGCTGTCAGAGCCTCGTGGCTTTGG
GAPDH	F	CACTCACGGCAAATTCAACGGCA
	R	GACTCCACGACATACTCAGCAC
