# Supplementary figures and images for: 21-O-Angeloyltheasapogenol E3, a Novel Triterpenoid Saponin from the Seeds of Tea Plants, Inhibits Macrophage-Mediated Inflammatory Responses in a NF-κB-Dependent Manner
Source: Mediators Inflamm. 2014 Nov 10;2014:658351. doi: 10.1155/2014/658351 (PMC4245502; doi:10.1155/2014/658351)

Supplementary Figure 1

NMR profile

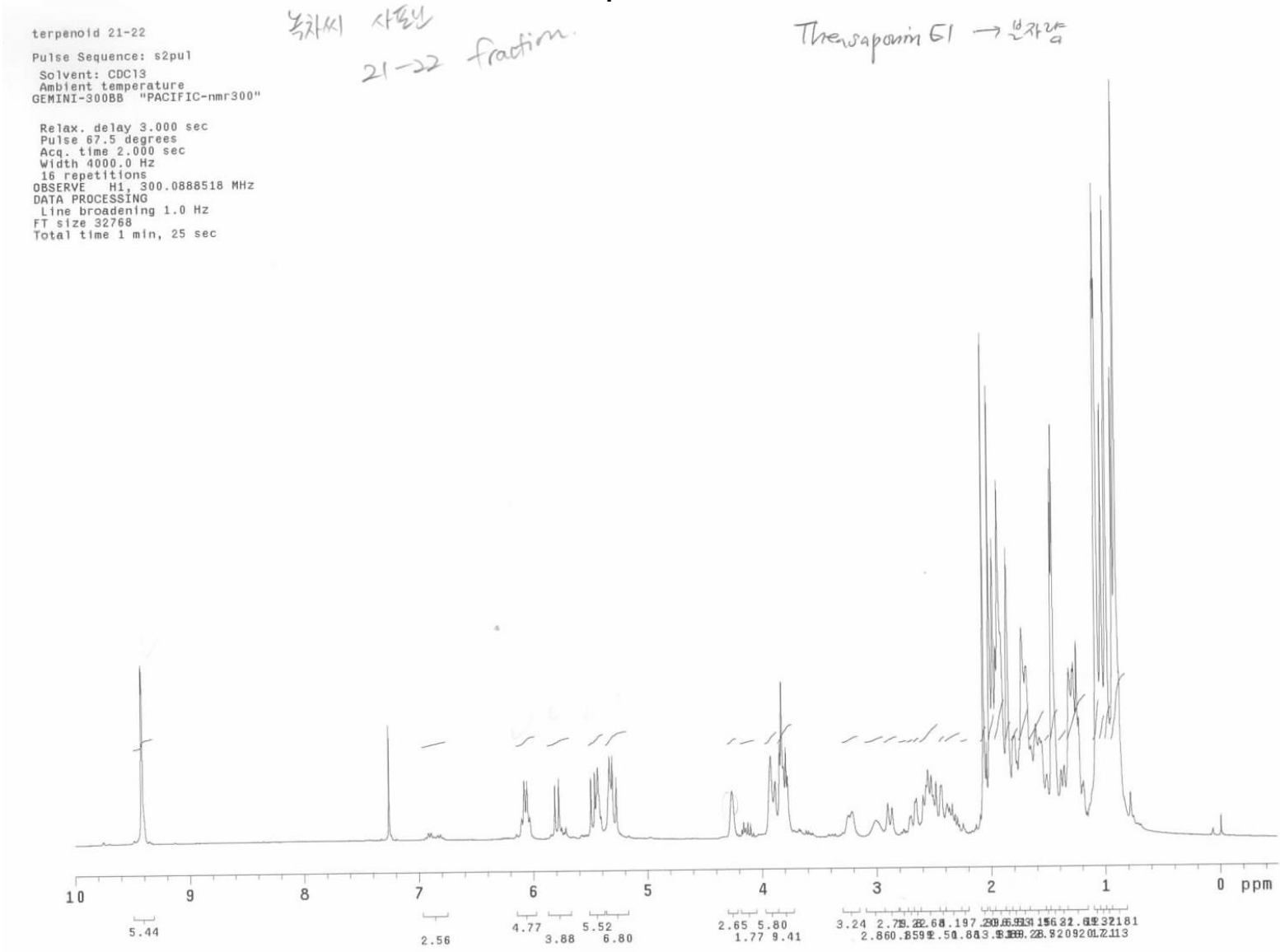

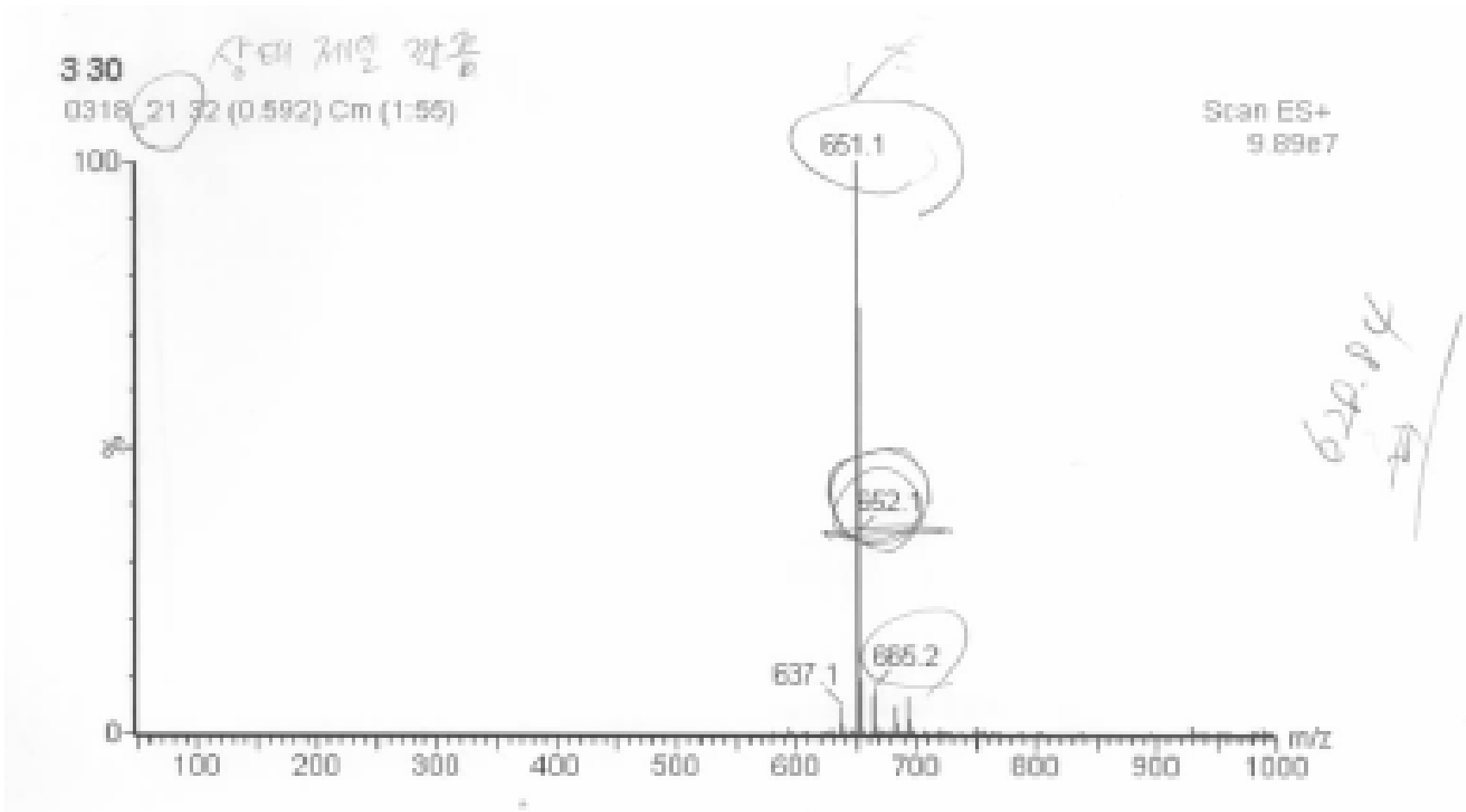

Supplement: Supplementary file 1 — Supplementary Figure 1: 1H NMR spectra were recorded on a Bruker Avance 300 (300 MHz) and Bruker DPX 400 (400 MHz). Chemical shifts are reported in parts per million (ppm) downfield relative to tetramethylsilane as an internal standard. Supplementary Figure 2: ESI-MS was measured on an Agilent 1100 LC/MS spectrometer with a Phenomenex Luna C18 analytical column (5 mm, 4.6 × 100 mm). [file 658351.f1.pdf]
